# Revisiting the Analysis of Radiative Mid-Range Wireless Link for Pacemakers

**DOI:** 10.3390/s22030947

**Published:** 2022-01-26

**Authors:** Ilkyu Kim, Bo-Hee Choi, Jeong-Hae Lee

**Affiliations:** 1 C4I Team, Defense Agency Technology and Quality, Jinju 52851, Korea; ilkyukim@gmail.com; 2 Department of Electronics and Electrical Engineering, Hongik University, Seoul 04066, Korea; passion86@naver.com

**Keywords:** radiative near-field, wireless link, implanted biomedical devices, pacemakers, asymptotic Friis formula, Chu formula, integral coupling formula

## Abstract

The development of a wireless link for biomedical applications requires an accurate estimation of the delivered power to implanted devices. In particular, a variety of mid-range applications in the biomedical area have gained significant attention. An appropriate method for the mid-range wireless link is required to implement a continuous wireless link through human tissue. Even though formulas used in this work are all based on previous works, this paper presents an implementation of the diverse formulas for the mid-range wireless link of an implanted antenna used for a pacemaker system based on the understanding on radiation properties varied with the distances from the antenna. The formulas based on input far-field data are successfully applied to compute the power transmission for the implanted devices, whose range includes radiative near-field and far-field regions. The wireless link for a pacemaker system is evaluated through using a patch antenna immersed with different depths of human tissue. A comparison of the computed and measured results shows an excellent agreement where the validity of the evaluation is demonstrated.

## 1. Introduction

The use of implantable medical devices (IMD) has led to the advancement of various medical treatments and diagnostic methodologies. In particular, pacemakers have received a considerable amount of interest for the patients suffering from abnormal heart rhythms. They have been used to successfully recover heart rhythms for the patients. The implantable medical devices, such as pacemakers, typically require wireless power transfer (WPT) as well as wireless data transfer [1]. The use of wireless devices is advantageous in contrast to the wire-based devices where battery life time and replacement are the biggest issues [2,3,4,5]. In spite of apparent benefits of the wireless devices, the implanted devices must meet strict requirements to provide the continuous wireless link with external devices. Considering low available power for the implanted devices, an accurate estimating method of the wireless link is required [6,7,8]. A variety of techniques based on inductive, capacitive, magnetic, mid-field, far-field, acoustic, and optical link have been investigated for WPT applications [9,10,11,12]. In particular, mid-range applications made in the radiative near-field or far-field region have gained significant attention. The traditional WPT operating in the VHF band has focused on a non-radiative near-field region, and the use of high-frequency applications in recent years permits the use of WPT systems realized in a radiative near-field region, which are known as “radiative mid-range” [13,14,15,16,17,18,19,20,21]. The mid-range applications allow the device to communicate through using the radiation characteristics of two antennas. Several techniques have been studied for the electromagnetic characteristics of various implanted antennas, including an interaction between the implanted antenna and the external antenna [22,23,24,25,26]. The ray tracing methods, such as geometrical optics (GO) and uniform theory of diffraction (UTD), are suitable for computing the electrically large objects. The methods have been used for the investigation on the outdoor point-to-point link, and have recently demonstrated their applicability to the case of body centric wireless link [22]. A finite-difference time-domain (FDTD) is useful for the study on electromagnetic problems with inhomogeneous media [23,24]. The accuracy and efficiency of the method has been investigated for the diverse biomedical devices. However, the FDTD method has been limited to the short wireless link in order to meet the specific discretization of unit cell with allowed computing capability. Meanwhile, the method of moment (MoM) provides a degree of freedom to diverse locations of biomedical devices [25,26]. The method has been applied to the calculation of the on-body wireless link; however, for the case with inhomogeneous and lossy media, the use of the MoM might be less efficient than using other methods. Even though those methods have been widely used in the study of the mid-range wireless link in biomedical areas, electromagnetic analysis of antennas in the human body usually requires intensive computation. Therefore, an efficient method needs to be employed in order to study diverse cases of wireless links in a limited time. The Friis formula, based on plane-wave characteristics, is computationally advantageous for the wireless link in the far-field region [27]. A variant of the Friis formula and integral coupling formula have been introduced to compute the wireless link in the radiative near-field region [28,29,30,31]. In the radiative near-field region, in contrast to the far-field region, broadened radiation patterns with reduced gains are observed. The ability to accurately estimate the changes of the radiation in a proximity distance is becoming more critical. The Friis formula with correction terms has been successfully utilized to estimate the wireless link in the Fresnel region [27,28,29]. In this formula, correction term as a function of far-field gain has been successfully applied to characterize the radiation properties in the Fresnel region. An approach to the generalization of the Friis formula based on the fundamental Gaussian beam has been proposed by Chu [31]. While the formulas are related to the rapid calculation based on the simple far-field gain, the integral coupling takes advantage of the fast Fourier transform (FFT) based on two vector far-field patterns [32,33,34,35,36,37]. This feature allows diverse scenarios of the wireless link to be easily computed from far-field patterns obtained from measurements or full-wave simulations. The effectiveness of the formula has been demonstrated through various cases of the power transmission in a free-space and more complex circumstances containing dielectrics between two antennas [36]. Recently, the formula has been applied to the wireless link for implanted biomedical devices [10]. Even though there are several recent advancements for the mid-range biomedical implanted devices, relatively little attention has been paid for accurately and efficiently analyzing the mid-range wireless link. Moreover, comprehensive evaluations based on diverse techniques using the radiative near-field characteristics have been rarely reported.

In this paper, the radiation characteristics around the implanted antenna are studied, and several techniques based on the far-field radiation characteristics are revisited and its applications to the wireless link for the implanted antennas are presented. The mid-range radiation characteristics are studied through the near-field distribution where the antenna is immersed with different depths of human tissue for pacemaker application. In the following section, the phenomenon of the reduction in the far-field gain, known as “gain reduction factor”, is used to describe the radiation characteristics in the radiative near-field region. This work provides an overview of important features for the several techniques used in the mid-range wireless link: (i) asymptotic Friis formula, (ii) Chu formula, and (iii) integral coupling formula. The detailed procedure to obtain the wireless link is discussed, and the formulas are applied to the various cases of the biomedical wireless link. In order to include all near-field wireless links, this work revisits the two-port network for the investigation of inductive coupling where the wireless link is made in a reactive near-field region.

## 2. Materials and Methods

The derivation of the related formulas, applications, and measurements have been profoundly discussed in [27,28,29,30,31,32,33,34,35,36,37]. The formulas based on the available far-field data are appropriate to obtain the power transmission between two antennas. It is demonstrated that the formulas allow for the accurate and efficient computation of the power transmission in a free-space or in a complex environment with the existence of dispersive dielectrics. In this section, the detailed procedure, to obtain the power transmission for the implanted antenna in biomedical applications, is presented. In order to discuss the important features of the related formulas, the radiation pattern needs to be characterized in terms of various locations from the antenna. In this section, the key features of the radiation characteristics and their interactions to obtain the power transmission are discussed. 

### 2.1. Asymptotic Form of Gain Reduction Factor

The radiation characteristics can be varied from the near-field region to the far-field region of the antenna of interest. There are two categories in the near-field region: reactive near-field region and radiative near-field region. The reactive near-field region is known as non-radiative near-field region or inductive near-field region. The boundary to distinguish reactive near-field from radiative near-field is known as λ/2π for an electrically small antenna [38]. This paper revisits the formulas that are effective in the mid-range region, including the radiative near-field and far-field region. The distinctive radiation characteristics in near-field and far-field regions are investigated through an example of a large aperture antenna, represented by a reflector antenna with a radius of 10 λ. 

**Figure 1:**
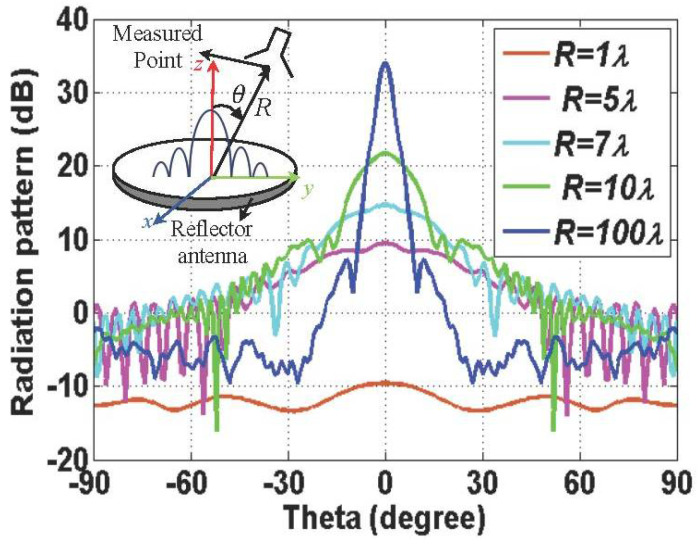
Varied radiation patterns of the parabolic reflector antenna with a radius of 10*λ* at the different positions of the non-radiative near-field region and the radiative near-field region.

Figure 1 describes the variation of the H-plane radiation pattern obtained from full-wave simulation, FEKO. The H-plane radiation pattern shows that there is a rare radiating part close to the antenna; as the distance increases, the radiation towards a boresight direction appears, and, finally, a uniform far-field pattern is created. For the on-axis direction, as the distance from the antenna increases, the magnitude of the power densities oscillates and then monotonically decreases, as presented in the previous work [29,33,36]. The effective wavelength $\lambda_{e}$ in the biological tissue presented in [7] can be defined as

(1) λe=λ0Re[εr−jσwε0]

where $\lambda_{0}$ and $\varepsilon_{0}$ are wavelength and permittivity in a free space, respectively, and $\varepsilon_{r}$ and σ  are relative permittivity and conductivity of the biological tissue. The surrounding environment for the implanted antenna is selected as the human skin, where its electrical properties are summarized in Table 1. The effective wavelength $\lambda_{e}$ in the human skin is 93.6 mm, from which the thickness of the human skin of 4 mm and 8 mm can be estimated as 0.043$\;\lambda_{e}$ and 0.085$\;\lambda_{e}$, respectively. The electrical distance from the implanted antenna can be reduced based on the $\lambda_{e}$, compared to the one in a free space. The effective wavelength is negligible in the far-field region, while it affects the electrical distance in the close part of the radiative near-field region and the reactive near-field region. The boundary between the radiative near-field and far-field region is suggested as 2*D*^2^/λ or 2λG/π^2^ [29]. The different radiation characteristics in each region can be applied to the derivation of the gain reduction factor γ, which is defined as

(2) $$\gamma \left(\Delta \right)=\frac{G_{F}\left(\Delta \right)}{G}=1-\alpha \Delta^{-2}$$

where the *G* and *G_F_* are the far-field gain and Fresnel gain of the antenna, respectively, and the coefficient *α* is related to the rate of the reduction in the far-field gain *G*, which is found to be *α* = 0.066. The normalized distance Δ can be defined as

(3) $$\Delta =\{\begin{array}{c} \frac{R}{2D^{2}/\lambda }\;\left(\mathrm{Traditional}\;\mathrm{definition}\right)\\ \frac{R}{2\lambda G/\pi^{2}}\;\left(\mathrm{Proposed}\;\mathrm{definition}\right)\; \end{array}$$

In this paper, the gain reduction factor with the proposed separation distance integrated with the Friis formula is used to enhance the accuracy in the radiative near-field region.

### 2.2. Mid-Range Wireless Link Formulas 

This subsection presents appropriate methods for calculating the mid-range wireless link and important features of the different techniques. Three different methods used in the evaluation include (i) asymptotic Friis formula, (ii) Chu formula, and (iii) integral coupling formula. The first two formulas based on the far-field gain are advantageous in providing an almost instant calculation. However, the drawbacks include the limitation of the possible scenarios and the slight loss of the effective range, compared to other methods. The integral coupling formula utilizes the FFT of the far-field data with magnitude and phase. Even though relatively complex far-field data are required, the applications of the formula, including various case studies, are very wide. The three different methods are all accurate and time-efficient, even when not taking the recent advancement of computing capabilities into consideration. 

#### 2.2.1. Asymptotic Friis Formula 

**Figure 2:**
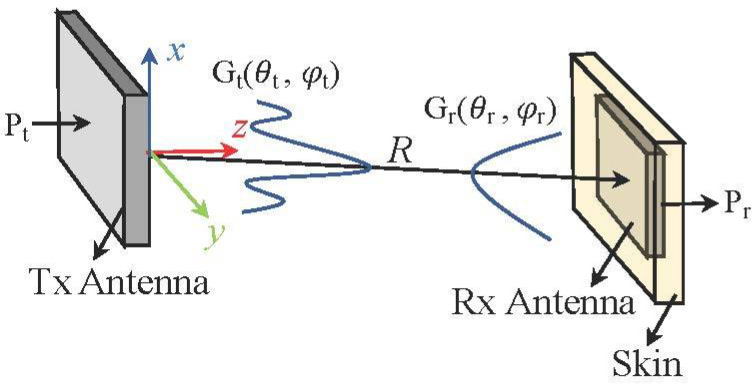
Configuration of the power transmission between a Tx antenna and a Rx antenna.

The Friis formula, derived from plane-wave characteristics, has been widely used for the long-range wave propagations [27]. A configuration of the power transmission between two antennas is depicted in Figure 2. A ratio between transmitted power $P_{t}$ and received power $P_{r}$ between two antennas can be obtained using the Friis formula, as follows:

(4) $$\;\frac{P_{r}}{P_{t}}={\left(\frac{\lambda }{4\pi R}\right)}^{2}G_{t}\left(\theta_{t},\varphi_{t}\right)G_{r}\left(\theta_{t},\varphi_{t}\right){\left|{\hat{\rho }}_{t}\cdot {\hat{\rho }}_{r}\right|}^{2}$$

where G(θ,φ) is the far-field gain in terms of angular variables of  (θ,φ), and *R* is the separation distance between two antennas. $\hat{\rho }$ is the polarization vector. The subscripts *t* and *r* represent the transmitting and receiving antenna, respectively. Although it predicts well in the far-field region, a comparison between the Friis formula and measurements imply that the Friis formula is inaccurate in a short distance [28,29]. The discrepancy in the radiative near-field region is owing to the plane-wave approximation that is suitable for the far-field region. The Friis formula can be incorporated with an asymptotic correction term for accurately calculating the mid-range wave propagation. The asymptotic term, as a function of distance *R* and far-field distance 2*D*^2^/λ, has been used to include the gain reduction effect in the radiative near-field region. The problem of the formula is the intense computation of the gain reduction factor required for each antenna. It has been found in [29] that the gain reduction factors tend to converge through taking the slightly modified form of the asymptotic term presented in Equation (3). The modification is beneficial in terms of its applicability to a variety of antennas with rapid computations. The use of the asymptotic expression, however, requires slightly larger minimum ranges where all of the radiative near-field region is not covered. The Friis formula with the gain reduction factors γ can be defined as

(5) $$\frac{P_{r}}{P_{t}}=\frac{\lambda^{2}}{16\pi^{2}R^{2}}G_{t}\gamma_{t}\left(R\right)G_{r}\gamma_{r}\left(R\right)$$

In order to include both cases of low and high gain antennas, the switching function *F*(*G*) is incorporated with the gain reduction factor  γ. The gain reduction factors of the transmitted antenna $\gamma_{t}\;$ and received antennas $\gamma_{r}$ can be defined as

(6) $$\gamma_{t}=1-\alpha_{E}F\left(G_{t}\right){\left(\frac{R}{2\lambda G_{t}/\pi^{2}}\right)}^{-2},\;\;\gamma_{r}=1-\alpha_{E}F\left(G_{r}\right){\left(\frac{R}{2\lambda G_{r}/\pi^{2}}\right)}^{-2}$$

$\mathrm{where}\;F\left(G\right)=2.5-\frac{a}{\pi }\cdot \arctan\left[c\cdot \left(G-b\right)\right]\;\mathrm{and}\;\alpha_{E}=$ 0.066.

In this work, the parameters, *a* = 3, *b* = 5, and *c* = 1, are selected for smooth switching. The other technique based on the Gaussian beam has been applied to both the radiative near-field region and far-field region. It is worth noting that *G_t_* and *G_r_* included in the correction term are substituted with the directivity of the transmitting and receiving antenna, respectively. 

#### 2.2.2. Chu Formula

An approximation of the Friis formula has been derived based on the fundamental mode of Gaussian beams, which is called “Chu formula”. The formula is a function of the distance *R* and effective aperture area *A_e_*, which is also relevant to the far-field gain *G*. The Gaussian beam is used to include both approximation in the far-field region and gain reduction effect in the radiative near-field region.

(7) $$\begin{array}{ll} & \frac{P_{r}}{P_{t}}=\frac{\lambda^{2}}{16\pi^{2}R^{2}}G_{t}G_{r}\\ \times & \frac{1}{1+\left(\lambda^{2}/\left(16\pi^{2}R^{2}\right)\right){\left(\left(\sqrt{F\left(G_{t}\right)}G_{t}+\sqrt{F\left(G_{r}\right)}G_{r}\right)/2\right)}^{2}} \end{array}$$

In contrast to the separable asymptotic gain reduction factors for transmitting and receiving antennas, the formula includes a combined form of the gain reduction factors of the two antennas. It is worth noting that the switching function *F*(*G*) is incorporated with the antenna gain in the correction term, except for the Friis formula. 

#### 2.2.3. Integral Coupling Formula

In the formula, a fast Fourier transform (FFT) has been used to obtain the near-field coupling from both the magnitude and phase of the far-field pattern [32,33,34,35,36,37]. The requirements of the formula are the complex far-field pattern and the antenna placements of both the on-axis and rotational scenarios. The formula requires the discretized sampling and implementation of the integral based on the FFT method. The use of the FFT method makes it possible to increase the computational efficiency, compared to a Fourier transform. The far-field patterns obtained at the phase center of an antenna are an important requirement for the FFT calculation. The first procedure is to transform the origin of the far-field pattern obtained from simulation or measurement into the global coordinate system. Eulerian angle transformation is used to allow origins of the far-field patterns to be located in the global coordinate system. The two-dimensional interpolation technique is used to obtain the complex far-field patterns of two antennas at sample points of the discretization. A distribution of far-field patterns confined in the rectangular plane provides the spatial bandwidth of the FFT. The sampling frequency is determined by the bandlimited characteristics using Nyquist theorem, and bandlimits the transverse displacement between two antennas.

(8) $$f_{s}=2\kappa \times \left(D_{t}+D_{r}\right)$$

where  κ is the oversampling ratio. The summation form of the coupling quotient presented in [33] can be defined as

(9) $$\begin{array}{ll} \frac{{b^{\prime }}_{0}}{a_{0}}\left(\vec{R}\right)= & -\frac{C}{k}{\left(\Delta k\right)}^{2}\\ & \times \underset{m}{\sum }\underset{n}{\sum }\frac{{\vec{f}}_{TX}\left(k_{x}^{mn},\;k_{y}^{mn}\right)\cdot {\vec{g}}_{RX}\left(k_{x}^{mn},\;k_{y}^{mn}\right)}{k_{z}^{mn}}\times e^{i{\vec{k}}^{mn}\cdot \vec{R}} \end{array}$$

where $\;{\vec{f}}_{TX}\left(k_{x}^{mn},\;k_{y}^{mn}\right)\;\mathrm{and}\;{\vec{g}}_{RX}\left(k_{x}^{mn},\;k_{y}^{mn}\right)$ are 3D vector far-field patterns of the transmitting and receiving antennas, respectively, and $k=\hat{x}k_{x}^{mn}+\hat{y}k_{y}^{mn}+\hat{z}k_{z}^{mn}$ and $\Delta k=\frac{2\pi }{f_{s}}$ = $\frac{\pi }{\kappa \left(D_{t}+D_{r}\right)}\;$. $a_{0},b_{0}$ represent amplitudes of input wave to transmitting and output wave from receiving antenna, respectively, where there is a relationship such as $S_{21}={\left|{b^{\prime }}_{0}/a_{0}\right|}^{2}$. The impedance mismatch term C can be defined as

(10) $$\;C=\frac{Z_{FL,\;RX}}{Z_{0}}\frac{1}{\left(1-\Gamma_{0,\;\;Tx}\Gamma_{0,\;\;Tissue}\right)}\frac{1}{\left(1-\Gamma_{0,\;\;Rx}\right)}$$

where $Z_{FL,\;RX}$ and $Z_{0}$ are the feedline impedance of the receiving antenna and intrinsic impedance in the air, respectively, and $\Gamma_{0,\;Tx},\;\Gamma_{0,\;Tissue},\;$ and $\Gamma_{0,\;Rx}$ are reflection coefficients of the transmitting antenna, the human tissue and the receiving antenna, respectively. It is worth noting that the Equation (9) has $e^{-i\mathrm{wt}}$ time dependence in free space, and multiple interactions are ignored.

With an effort to extend the effective range, it is demonstrated that the far-field approximation to the integral coupling is well converged into the Friis formula. A valid range of the integral coupling, therefore, includes radiating both the near-field region and far-field region.

## 3. Results

The radiation characteristics of an antenna inside the human tissue are studied through the near-field distribution, where the antenna is immersed with different depths of human tissue for the pacemaker application. In the following subsection, the phenomenon of the reduction in the far-field gain, known as “gain reduction factor”, is used to describe the radiation characteristics in the radiative near-field region. Based on the understanding of the gain reduction factors, the related formulas are presented to predict the power transmission made in the radiative near-field region. 

### 3.1. Evaluation of Gain Reduction Factor

**Figure 3:**
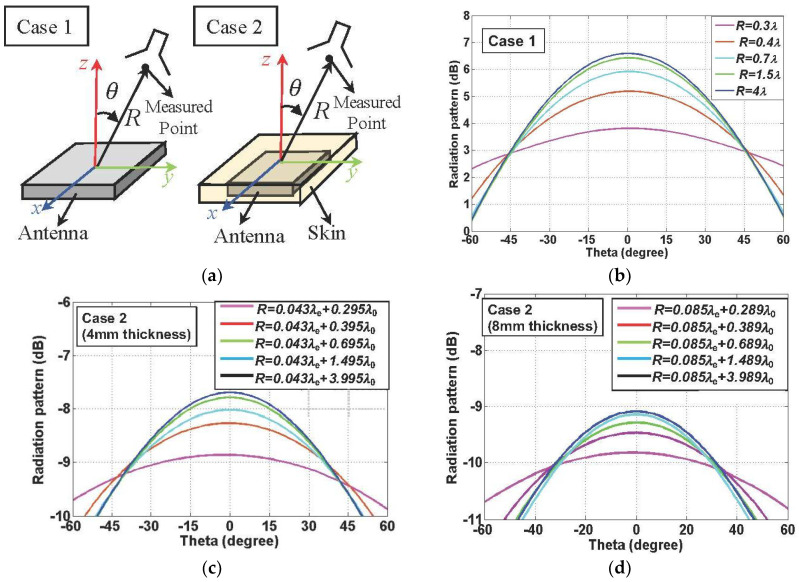
(**a**) Description of the evaluated cases, (**b**) simulated radiation patterns for the reader patch antenna outside the human body, (**c**) simulated radiation patterns for the implanted patch antenna immersed with 4 mm thickness of the human skin, and (**d**) simulated radiation patterns for the implanted patch antenna immersed with 8 mm thickness of the human skin.

A useful representation of the reduced antenna gain in the radiative near-field region is the gain reduction factor. The effects of the significant decrease in the near-field power transmission indicate that the reduction in antenna gain exists in a proximity distance. The near-field distribution of a patch antenna inside the human tissue is presented to examine the gain reduction effect of the biomedical devices. The radiation characteristics are investigated for the patch antenna immersed with different depths of the human tissue, whose specification was presented in [10]. The size of the implanted patch antenna is 20 cm × 20 cm, which is located between two 3.4 mm thick substrates with *ε_r_* = 10.2. The size of the reader patch is 40 cm × 40 cm, which is printed on 3.4 mm thick substrates with *ε_r_* = 2.2. As shown in Figure 3, the radiation pattern obtained from the full-wave simulation is varied depending on different distances *R* between the implanted antenna and the measured points. The radiation patterns, changed from the flat radiative pattern to the directive far-field radiation pattern, are described. The radiation patterns are obtained with different thicknesses of the human tissue, which are set as 4 mm and 8 mm. Since the thickness of the human tissue is comparable to the distance *R* for the case of small *R*, the distance *R* is measured as the wavelength in the air, *λ*_0_ and the one in skin tissue, *λ*_e_. The broadside gains at the different positions are used to derive the curve of the gain reduction factor. The gain reduction factor is obtained using the asymptotic formula presented in [29]. The modified antenna gain is applied to increase the accuracy of the formula in [29]. The modified gains for the reader patch antenna and other patch antennas immersed with different depths of skin are obtained through using the switching function *F*(*G*). It is observed that the gain reduction for the case of the reader patch antenna is greater than the other case of the implanted antenna. It is attributed to the fact that the gain reduction factor with the higher far-field gain typically produces the larger reduction in the antenna gain at the same distance *R*.

**Figure 4:**
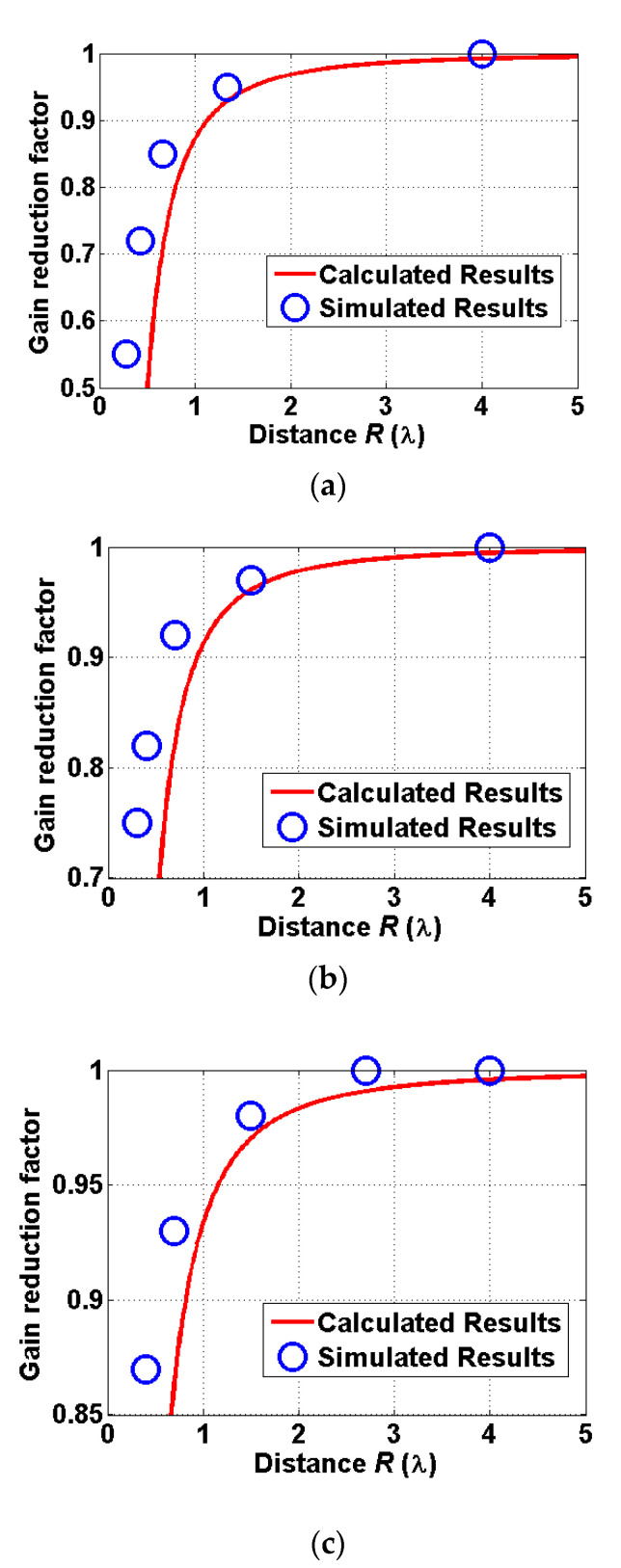
A comparison of calculated and simulated gain reduction factors for: (**a**) the reader patch antenna outside the human body, (**b**) the implanted patch antenna immersed with 4 mm thickness of the human skin, and (**c**) the implanted patch antenna immersed with 8 mm thickness of the human skin.

A comparison between calculated and simulated gain reduction factors is provided in Figure 4, which shows a good agreement between those two results except for a very close distance. For a very close distance which is less than 0.5*λ*, the gain reduction factor rapidly decreases due to the failure of the quadratic form of gain reduction factor in the close proximity. The power transmission based on the gain reduction factor is investigated in the following subsection.

### 3.2. Wireless Link Analysis Based on Related Formulas 

The main purpose of this section is to examine the power transmission to the implanted antennas for the pacemaker application. A variety of formulas discussed in the previous section are applied to the case of the near-field and the far-field wireless link. Based on the discussion of the radiation characteristics for different positions, biomedical applications of the formulas are provided in this section. The formulas have been applied to the on-axis and rotational scenarios which are commonly used in biomedical applications.

#### 3.2.1. On-Axis Scenarios 

**Figure 5:**
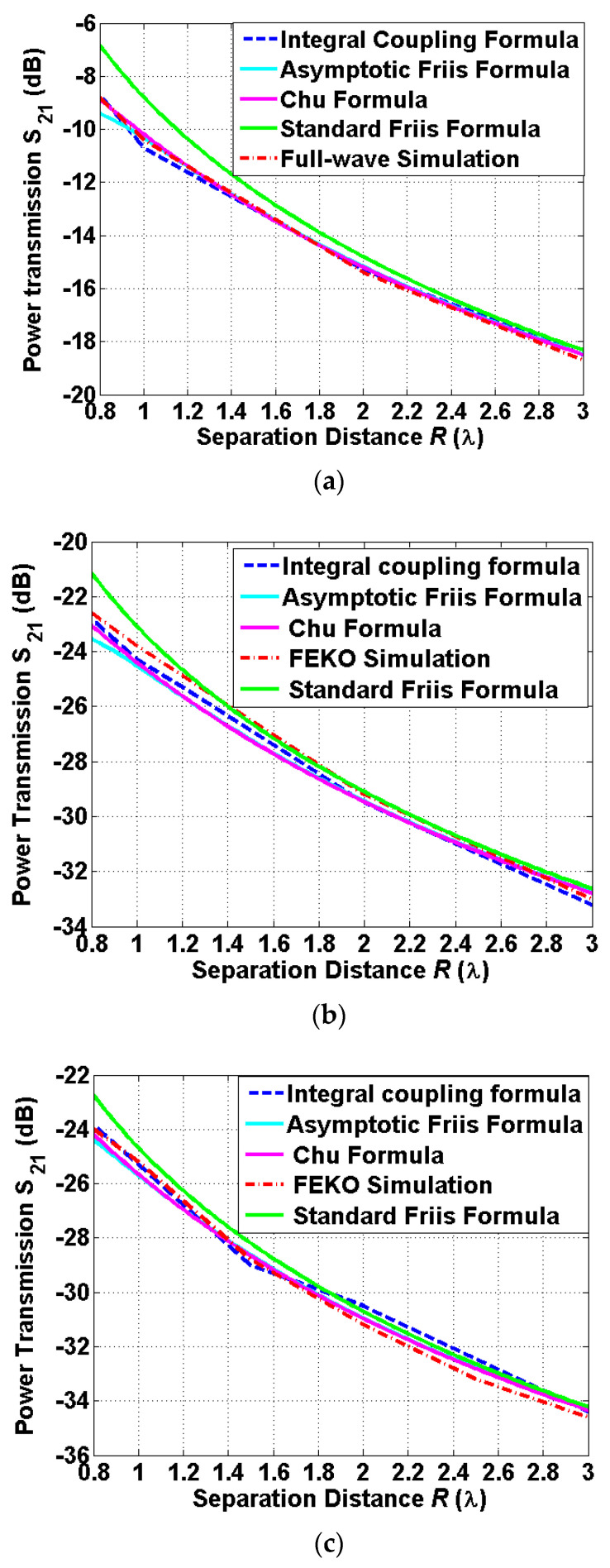
A comparison of the power transmissions obtained from the related formulas and the full-wave simulation FEKO at 400 MHz between: (**a**) the two identical reader patch antennas outside the human body, (**b**) the reader patch antenna outside the human body and the implanted patch antenna immersed with 4 mm thickness of the human skin, and (**c**) the reader patch antenna outside the human body and the implanted patch antenna immersed with 8 mm thickness of the human skin.

The integral coupling formula has been used to successfully evaluate the biomedical wireless link presented in [10]. The use of the diverse techniques is desirable to meet the requirement of establishing the biomedical wireless link where an efficient and accurate estimation is preferred. Other techniques used in this paper, compared to the previous work [10], are the asymptotic Friis formula and the Chu formula. The power transmission between two antennas is evaluated for the on-axis scenario where the implanted antenna is fixed while the reader antenna moves along the *z*-axis. The far-field gains and directivities applied to the formulas are provided in Table 2. The far-field gains are used to estimate the coupling level, including losses due to the implantation inside the human body, while directivities are used to calculate the gain reduction factors. The modified gain based on the switching function is used to determine both the gain reduction factors included in the asymptotic Friis formula and the Chu formula. Figure 5 shows a comparison for the related formulas and full-wave simulation FEKO. It is observed that the related formulas provide an excellent agreement with the results of full-wave simulation FEKO, except for the Friis formula. The Friis formulas provide maximum deviations (enhancements) of 1.7~2.5 dB from other methods. The case of the free-space power transmission provides more deviation (enhancement) compared to the case of the implanted antennas, since a relatively high gain of the antenna outside the human body produces more gain reduction in the near-field region. It is observed that the level for the case with 4 mm thickness is roughly 1.3–1.7 dB higher than the level of the case with 8 mm thickness. It is observed that the deviation from the Friis formula agrees well with the reduction in the antenna gain presented in Section 2. It is worth noting that the asymptotic Friis formula tend to diverge from a group of curves at 0.8*λ*, since the gain reduction factor is starting to decrease rapidly, as discussed in Section 2. 

#### 3.2.2. Rotational Scenarios

**Figure 6:**
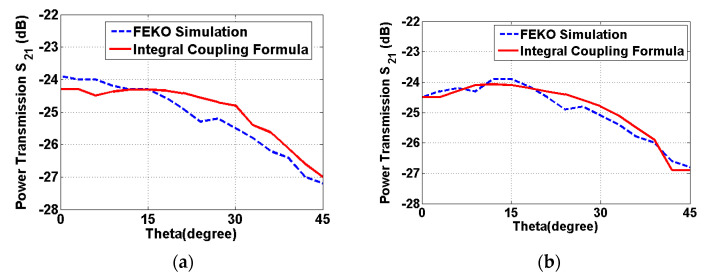
A comparison of the power transmission obtained from the FEKO simulation and the integral coupling formula at 400 MHz within a valid angular range at *R* = 1*λ* between: (**a**) the reader patch antenna and the implanted patch antenna immersed with 4 mm thickness of the human skin and (**b**) the reader patch antenna and the implanted patch antenna immersed with 8 mm thickness of the human skin.

The applicability of the formulas is examined for the case of the rotational scenario. The power transmission is evaluated for the case in which the implanted antenna is fixed while the other one rotates with an angle θ. The integral coupling formula is more flexible for the estimation of the power transmission due to the movement of the antenna. The integral coupling formula can be used to compute the power transmission in rotational scenarios, while the formulas originated from the Friis formula are restricted to accurately estimate the rotational scenarios. The integral coupling formula was primarily used for the evaluation of the rotational scenarios. The separation distance was set as 1λ and the rotation angles of the reader antenna varies from 0° to 45°, which are slightly wider than the valid angle presented in [34]. A comparison of the integral coupling formula and the full-wave simulation tool FEKO is shown in Figure 6. The similarity of the two graphs is observed in terms of the shapes with the different rotation angles. A maximum deviation between the integral coupling formula and the full-wave simulation FEKO is approximately 0.8 dB. The integral coupling formula demonstrates its applicability to the diverse scenarios which are widely used in the biomedical applications. 

### 3.3. Measurement

**Figure 7:**
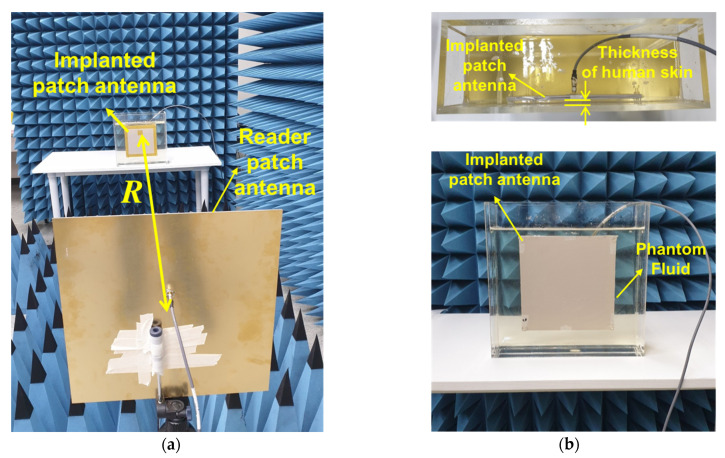
Photographs of the measurement setup for: (**a**) the on-axis power transmission between the implanted patch antenna and the reader patch antennas outside human body at 400 MHz, and (**b**) the patch antennas located in the tank filled with phantom fluid.

**Figure 8:**
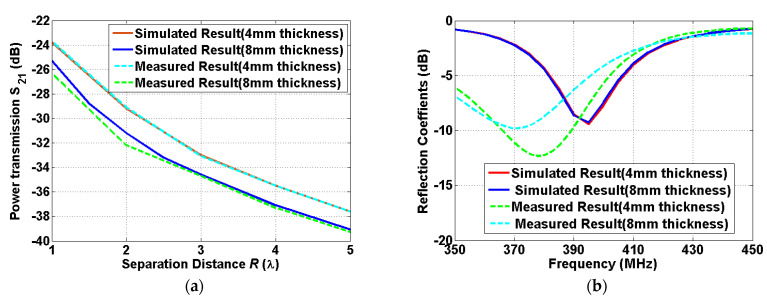
A comparison of the simulated results and the measured results for: (**a**) the power transmission between the reader patch antenna outside the human body and the implanted patch antennas with different thicknesses of the human skin at 400 MHz, and (**b**) the impedance matching of the implanted antennas with different thicknesses of the human skin.

The anechoic chamber measurements were performed to verify the validity of both the computed and simulated results. The setup for the indoor measurement is shown in Figure 7. A phantom fluid that provides the characteristics of the human skin was applied to the measurements. The product (SKIN350-500V2) manufactured by Schmid & Partner Engineering AG company, Zurich, Swiss is used. An acrylic tank is used to contain the fluid for the implanted patch antenna. The size of the acrylic tank is set as (height × length × width) = (31.6 cm × 31.6 cm × 10 cm), which can be filled with one liter of the fluid. The implanted patch antenna is situated inside the fluid, while the reader patch antenna moves along the boresight direction of the two antennas. An important aspect of this work is the investigation of wireless power transmission to implanted antennas which are immersed with different depths of 4 mm and 8 mm. The depths of the human skin were determined by measuring the distance between the implanted antenna and the wall of the tank. The power transmission was measured using a vector network analyzer by varying the separation distance from *R* = 1 *λ* to *R* = 5 *λ*. A comparison of the simulated results and measured results is shown in Figure 8. 

An excellent agreement between the simulated and measured results is observed for the different depths of the human skin. It is noted that the simulated coupling level at the shortest distance is slightly smaller than the one of Figure 8, since the shortest distance of *R* = 1*λ* for the measurement is greater than the one of *R* = 0.8*λ* for the simulation. For the impedance matching, the simulated and measured results are similar in terms of the magnitude of the reflection coefficients, while a 3~5% shift of the center frequency between the simulated and measured results is generated. The discrepancy between the simulated and the measured one might be attributed to the use of a relatively smaller size of the tank than the human tissue model used in the simulation. The effect from the dispersive phantom fluid can be reduced due to the limited size of the tank, which results in higher resonant frequency than the full-wave simulation. The measured results provide a good agreement with the simulated results for both the power transmission and impedance matching. 

## 4. Discussion

This paper presents biomedical applications of the formulas to compute the mid-range wireless link in the radiative near-field region. The radiation characteristics of the implanted antennas in the radiative near-field region are studied, and the related gain reduction factor is derived. The step by step procedures from the radiation characteristics to the analysis of the wireless link are presented. The formulas require the far-field information to compute the coupling between two antennas in the radiative near-field region. An overview of the different formulations is summarized in Table 3. The important aspects of the formulas are presented in terms of scenarios, computing time, input data, and effective regions. The case of the full-wave simulation FEKO is classified into the MoM method. The formulas were used to successfully compute the power transmission for the implanted patch antennas immersed with different depths of the human skin. A good agreement with the results obtained from the full-wave simulation FEKO is observed, and an enhancement of 1.7~2.5 dB, with respect to the Friis formula, was obtained. For the rotational scenario, a reasonable agreement with the results obtained from full-wave simulation FEKO was obtained. The validity of the evaluation was verified using the indoor measurement, and a good agreement between the simulated and the measured results was obtained. This work presents effective methods in the radiating near-field region; however, an uncovered proximity distance still exists, especially at lower frequency bands. In order to include the inductive coupling in the reactive near-field region, an example of the coupling at 400 MHz between a coil outside the human body and a loop antenna inside the human body is presented in Appendix A. The effective region of the proposed formulas implies that the (physical) minimum range can be reduced at a higher frequency band, such as 2.4 GHz, for body area communication.

## Figures and Tables

**Table 1:** Electrical properties of human tissue (skin) at 400 MHz.

Tissue	Permittivity (*ε_r_*)	Conductivity (*σ*[S/m])	Mass Density (*ρ*[kg/m^3^])
Skin	46.74	0.69	1100

**Table 2:** Far-field gains of the patch antennas at 400 MHz.

	Patch Antenna in the Air	Implanted Patch Antenna (4 mm)	Implanted Patch Antenna (8 mm)
Far-field gains	6.8 dBi	−7.7 dBi	−9.1 dBi
Directivities	6.6 dBi	5.5 dBi	5.2 dBi

**Table 3:** Overview of the different techniques for the radiative near-field regions.

	Scenarios	Computing Time (One Position)	Input Data	Effective Region
Asymptotic Friis formula	On-axis scenario	Instant	Far-field gain	Fresnel region
Chu formula	On-axis scenario	Instant	Far-field gain	Fresnel region
Integral coupling formula	On-axis, off-axis, and rotational scenarios	few seconds	3D vector far-field pattern	Radiative near-field
The method of moments (MoM)	On-axis, off-axis, and rotational scenarios	20~25 min	EM antenna designs and geometries	Radiative and non-radiative near-field
Finite-difference time-domain (FDTD)	On-axis, off-axis, and rotational scenarios	40~45 min	EM antenna designs and geometries	Radiative and non-radiative near-field

**Figure A1:**
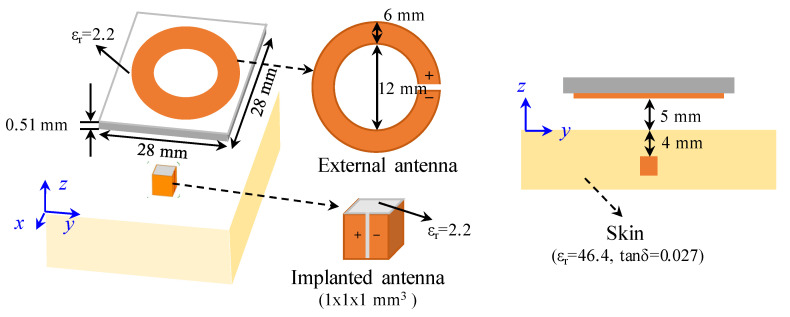
Description of the inductive coupling between the coil outside human body and the implanted loop antenna inside human skin.

**Figure A2:**
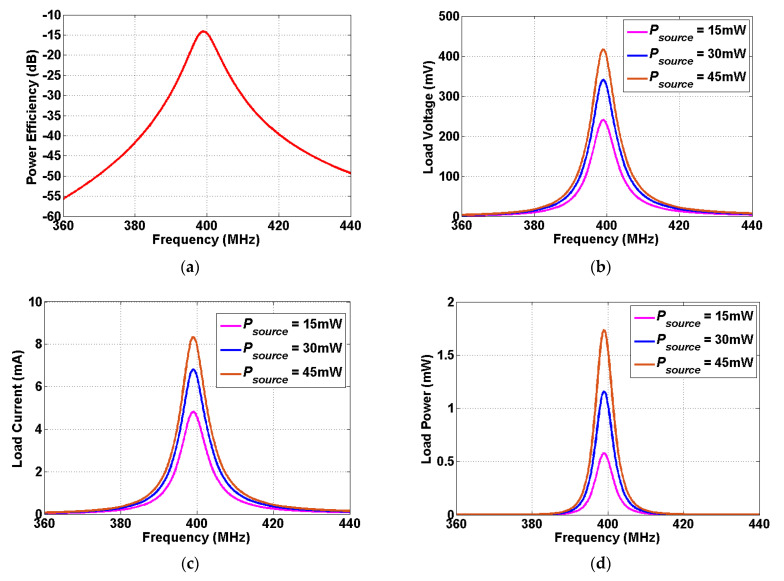
An inductive coupling between the coil outside the human body and the implanted loop antenna, and the simulated results for (**a**) power transfer efficiency, (**b**) voltage, (**c**) current, and (**d**) power in the load.

## Data Availability

Not applicable.

## Appendix A

In this Appendix, two-port network analysis ([39], Ch. 4.3) is revisited to estimate the power transfer efficiency for an inductive WPT system. The two-port network can be expressed as impedance parameters (Z-parameters). This technique, based on the circuit analysis, is useful to acquire the voltages, currents, and powers for a load of the receiving antenna. The purpose of this Appendix is to examine a power transfer efficiency between the coils outside the body and an electrically small loop antenna inside the human body. Figure A1 depicts the simulation setup of the inductive coupling between the small implanted antenna and the external antenna. The coupling in the reactive near-field region was evaluated at 400 MHz. The diameter of the external antenna is 24 mm, while the size of the implanted antenna is 1 × 1 × 1 mm^3^. The coupling between two antennas is expressed as the impedance parameters (Z-parameters) of the two-port network. The Z-parameters are obtained using a full-wave simulation tool, HFSS. Both load and source impedances are set as 50 Ω. The power transfer efficiency is obtained based on the simulated results of the Z-parameters and voltage, current, load power are derived based on the power efficiency. Figure A2 describes the power efficiency obtained from the full-wave simulation and corresponding voltage, current, and power delivered to the load. The distance between the coil outside the body and loop antenna inside the body is set as 9mm, and the loop antenna is immersed with a depth of 4 mm. The maximum power transfer efficiency is obtained as −15 dB, which is slightly higher than the one in the previous work ([39], Ch. 4.3). The magnitude of the power obtained in the load of the receiver is 0.57~1.74 mW when the power applied to the source is 15~45 mW. It is demonstrated that the impedance parameters based on the inductive coupling are effective for predicting the coupling in the reactive near-field region, as discussed in [40].
